# Management of radiation oncology patients with a pacemaker or ICD: A new comprehensive practical guideline in The Netherlands

**DOI:** 10.1186/1748-717X-7-198

**Published:** 2012-11-24

**Authors:** Coen W Hurkmans, Joost L Knegjens, Bing S Oei, Ad JJ Maas, GJ Uiterwaal, Arnoud J van der Borden, Marleen MJ Ploegmakers, Lieselot van Erven

**Affiliations:** 1 Catharina Hospital Eindhoven, Department of Radiation Oncology, Eindhoven, The Netherlands; 2 Netherlands Cancer Institute/Antoni van Leeuwenhoek Hospital, Department of Radiation Oncology, Amsterdam, The Netherlands; 3 Verbeeten Instituut, Department of Radiation Oncology, Tilburg, The Netherlands; 4 Jeroen Bosch Ziekenhuis, department of clinical physics, Hertogenbosch, The Netherlands; 5 Flevoziekenhuis, Department of Cardiology, Almere, The Netherlands; 6 Department of Radiation Oncology, University Medical Centre Groningen, Groningen, The Netherlands; 7 Department of Cardiology, Leiden University Medical Centre, Leiden, The Netherlands; 8 Dutch Society of Radiotherapy and Oncology (NVRO), Utrecht, The Netherlands; 9 Dutch Society of Specialists in Invasive Heart Stimulation (VITHaS), Utrecht, The Netherlands; 10 Dutch Society of Clinical Physics (NVKF), Utrecht, The Netherlands; 11 Order of Medical Specialists (OMS), Utrecht, The Netherlands; 12 Dutch Society of Cardiology (NVCC), Utrecht, The Netherlands

**Keywords:** Pacemaker, Implantable cardioverter defibrillator, Guideline, CIED, Risk management

## Abstract

Current clinical guidelines for the management of radiotherapy patients having either a pacemaker or implantable cardioverter defibrillator (both CIEDs: Cardiac Implantable Electronic Devices) do not cover modern radiotherapy techniques and do not take the patient’s perspective into account. Available data on the frequency and cause of CIED failure during radiation therapy are limited and do not converge. The Dutch Society of Radiotherapy and Oncology (NVRO) initiated a multidisciplinary task group consisting of clinical physicists, cardiologists, radiation oncologists, pacemaker and ICD technologists to develop evidence based consensus guidelines for the management of CIED patients. CIED patients receiving radiotherapy should be categorised based on the chance of device failure and the clinical consequences in case of failure. Although there is no clear cut-off point nor a clear linear relationship, in general, chances of device failure increase with increasing doses. Clinical consequences of device failures like loss of pacing, carry the most risks in pacing dependent patients. Cumulative dose and pacing dependency have been combined to categorise patients into low, medium and high risk groups. Patients receiving a dose of less than 2 Gy to their CIED are categorised as low risk, unless pacing dependent since then they are medium risk. Between 2 and 10 Gy, all patients are categorised as medium risk, while above 10 Gy every patient is categorised as high risk. Measures to secure patient safety are described for each category. This guideline for the management of CIED patients receiving radiotherapy takes into account modern radiotherapy techniques, CIED technology, the patients’ perspective and the practical aspects necessary for the safe management of these patients. The guideline is implemented in The Netherlands in 2012 and is expected to find clinical acceptance outside The Netherlands as well.

## Introduction

Pacemakers and ICDs may sustain damage during a course of radiation therapy. ICDs are devices that incorporate pacemaker functionality as well as the ability of producing a high voltage shock to terminate potentially lethal cardiac arrhythmias. Pacemaker and ICDs together are called Cardiac Implantable Electronic Devices (CIEDs). Guidelines for the management of patients receiving radiotherapy with a CIED [1-10] are not widely implemented [2,11]. The most cited guideline is the AAPM guideline originating from 1994 [10]. Since then, radiation therapy and CIED technology have changed significantly whereas the number CIEDs implanted and the number of patients receiving radiotherapy have increased tremendously. Radiation therapy is shifting from the use of primarily conventional techniques and conventional fractionations to IMRT and arc techniques and the use of more hypofractionated schedules. The higher fraction dose might lead to a potentially higher dose per fraction to a CIED. However, the use of more modern techniques has led to a reduced use of high energy photon beams, potentially leading to less dose to a CIED. Therefore, there is an urgent need for multidisciplinary evidence based practice guidelines for the management of patients receiving radiotherapy with CIEDs.

## Methods and materials

A multidisciplinary task group was organised to develop an evidence based guideline for the management of patients receiving radiotherapy with CIEDs. Major radiation modalities were taken into account; external photon and electron beams (up to 21 MeV). 60Co beams were not considered as they are not used anymore in The Netherlands. Also orthovoltage beams are seldom used in The Netherlands and if used outside the thoracic region, these were considered not to pose any problems. Electron beams at this energy or lower actually present a smaller problem than photon beams, as the neutrons production in these beams is much lower and neutrons actually seem to generate the majority of CIED defects. Electron therapy produces only 5% (at 15 MeV; [12]) to 20% (at 25 MeV; [13]) of the neutron dose equivalent per gray as photon therapy at the same nominal energy. This guideline is also applicable to patients receiving gamma-ray brachytherapy. Due to the rapid dose fall-off of this modality the CIED dose will generally be small. The lower energy spectrum of gamma brachytherapy implies a larger relative contribution of the photo-electric effect due to high Z materials in the CIED, and thus a localized higher dose to the CIED might be anticipated than the usually reported dose to water in surrounding tissue. However, as reports about brachytherapy use in this patient population did not reports any CIED problems yet, it was considered that this treatment modality could be handled equivalent to external beam photon therapy until more data will become available in the literature [14,15]. The specific influence of imaging techniques like CT, EPID and CBCT on CIED dose have not been described in detail within this guideline. Generally, the dose from these imaging techniques will be low. For example, Diederich *et al*. indicate an axial air kerma _CTDIair_ of 30–50 mGy for a typical multislice chest CT of an adult of average size [16]. For 4D-CT scans, the dose is typically in the order of 0.1-0.4 Gy while the dose from a kV-CBCT scan is typically much lower (10–80 mGy) [17]. The literature was first searched for guidelines and systematic reviews in the Cochrane Library and through SUMsearch. Thereafter, a Medline (OVID) search was conducted, which produced articles that were referenced in the previously found literature. This search was performed in May 2010, combining search terms “radiotherapy” or “radiation therapy” with “ICD” or “pacemaker” or “cardiac defibrillator”.

However, some more recent articles Based on this literature, the Dutch guideline was written. This article is a summary of the Dutch guideline which can be found at: http://www.kwaliteitskoepel.nl/kwaliteitsbibliotheek/uitgebreid_zoeken/radiotherapie-bij-patienten-met-een-icd-of-pacemaker.html. During the preparation of this guideline, the AAPM also recognised the value of renewing the guideline. A new Taskgroup (TG 203) was formed and it is expected that this guideline will be ready in 2014. One of the authors of the Dutch guideline is also a member of this AAPM Taskgroup.

### Literature review

An overview of some of the important papers on this topic is presented in Table 1 There still is a paucity of data and many papers are based on patient case reviews rather than large cohorts of patients or large numbers of CIEDs irradiated in vitro.

**Table 1:** **Overview of important in vivo and in vitro studies** (**with either a large number of CIEDs or interesting findings**, **excluding reviews**)

**Year**	**First author**	**Study type**	**Number of PM included**	**Number of ICD included**	**Intervention**: **Max Dose in study**(**Gy**)	**Number of defects**	**type of defect**
2010	Ferrara	Prospective in vivo	37	8	< 2,5	No defects	No defects
2010	Wadasadawala	Review+ 8 pacemakers	8	0	60	No defects	No defects
2009	Zweng	case report	1	0	0.11	1 [1] @ 0.11 Gy	runaway PM
2009	Gelblum	Retrospective in vivo	0	33	<3	1 [33] @3 Gy	Reset to factory settings
2008	Lau	case report	0	1	<0,15	1 [1] @ 0,15 Gy	electrical reset
2008	Kapa	in vitro research + in vivo retrospective	7 (in vivo)	20 (in vitro) 5 (in vivo)	< 4 (in vitro) unreported for in vivo	No defects	No defects, 4 devices relocated before RT
2008	Munshi [52]	case report	1		4.3	No defects	No defects
2007	Nemec	case report	0	1	< 6	1 [1] @ <1 Gy*	Runaway ICD
2005	Hurkmans [24]	in vitro research	19	0	< 120	14 [19] @ 120 Gy 1 [19] @ 20 Gy	Output, sense and communication
2005 2006	Hurkmans Uiterwaal [24]	in vitro research	0	11	< 120	11 [11]@120 Gy (irreversible) 4 [11]@ 0,5 Gy (minor defects)	To low shock energy, sensing and Battery charge time, erroneous VF or VT detection.
2004	John	case report	0	1	50	1 [1] @ not reported dose	shock impedance (coil failure)
2002	Mouton	in vitro research	96	0	200	4(96) @ 0,2 Gy 21(96)@2 Gy >0.2 Gy/min	8 defect modes described
2001	Niehaus	Review+ in vivo research	0	3	< 5	no defects	No defects
2000	Tsekos	case report	1	0	< 50	1 [1] @ not reported dose	Decrease of battery load
1994	Souliman	in vitro research	18	0	70	11 [18] @ 7,0 Gy 2 [18] @ 1,7 Gy 5 [18] @ 2,5 Gy	1) temporary change to interference or safety mode pacing lasting for the duration of the irradiation only [2] change to interference mode pacing—from which recovery may occur after reprogramming the pacemaker [3] severe damage
1994	Wilm [53]	in vitro research	20	0	300	2 [20] @ 10 Gy (complete defects)	Complete defect, decrease of pace amplitude, loss of telemetry
1991	Rodriguez	in vitro research	23	4	< 50	1 [23] @ 14 Gy 11 [23] sensitivity 9 [23] telemetry	sensitivity, telemetry and total defect

* estimated from the case report.

### CIED technology

Modern CIEDs contain Complementary Metal Oxide Semiconductor (CMOS) technology. The CMOS technology developed in the early nineties was more radiosensitive than the previously used bipolar transistors (TTL technology) [9]. However, CMOS technology has rapidly developed and currently is much more radioresistant. It is for example routinely used for aerospace applications requiring a tolerance up to several thousand Grays [18]. TTL CIEDs have not been implanted in recent decades so an assumption can be made that patients with CIEDs no longer carry such a device. Modern CIEDs, however, are still radiosensitive because of their increased circuit complexity, the ever decreasing power consumption and possibly the decreased dose attenuation of the CIED case. The leads are generally considered to be insensitive to radiation, although one case report claims irradiation-induced damage of the leads, leading to an observed shock coil failure [19].

### Pacemaker versus ICD

CIED manufacturers have diverging opinions on sensitivity of different CIEDs and advices for the management of patients with CIEDs during radiotherapy [2]. A guideline by Guidant published in 2003 stated that ICDs might be more sensitive because operating instructions are stored in RAM memory [20]. In a later (2008) publication by Boston Scientific (which incorporated Guidant in 2005) this difference is no longer cited, then stating that there is no safe lower dose limit and that some studies consider 2 Gy as the maximum allowable safe dose to a CIED [21]. CIED manufacturer Medtronic, although no supportive data are reported, claims that the dose tolerance is 5 Gy for pacemakers and 1–5 Gy for ICDs with the dose tolerance being specific to the type of ICD. Medtronic reports minor pacemaker damage above 5 Gy [22]. St. Jude Medical states the risk of effects on device operation increases with increasing cumulative radiation exposure and that no exact threshold for damage has been determined; the range has been “as low as” 20 Gy in some devices and as high as 150 Gy in others. In a table of defect frequencies, they do not make a distinction between pacemakers and ICDs [23].

### In vitro studies: ICDs in the direct beam

In all 11 ICDs placed in a direct 6 MV beam as studied by Uiterwaal et al., defects were observed [24]. These defects caused loss of pacing or rapid ventricular pacing, which in patients might lead to ventricular fibrillation. Four out of 11 ICDs incorrectly detected ventricular fibrillation or tachycardia, which would lead to inappropriate therapy, i.e., delivery of a high voltage shock.

### In vitro studies: Electromagnetic interference effects

Electromagnetic interference (EMI) may lead to inappropriate sensing of a myocardial potential, resulting in inhibition of the output, fixed rate pacing or reprogramming. These effects are mainly temporary or reversible. There are no published studies reporting serious problems near linear accelerators [10]. Electromagnetic fields around modern linear accelerators have decreased, reducing the concern to patients with a CIED [4,9,24-27]. Therefore, EMI does not seem to be of clinical relevance.

### In vitro studies: Dose rate effects

There is to our knowledge only one published study involving dose rate effects on CIEDs [28]. Of the 96 pacemakers studied, none showed any defects at a dose rate of 0.2 Gy/min. Only two devices had a defect below 1 Gy/min. Most of the first defects (78 out of 96) were observed at dose rates of 8 Gy/min or higher. The authors concluded that one could consider 0.2 Gy/min as the maximum acceptable dose rate. Some parts of the CIED, mainly the parts involved in rhythm sensing, reference voltage and physiologic sensing, are possibly sensitive to temporary interference from a high dose rate. This may inadvertently cause an ICD to deliver a shock, stop pacing, reset itself or display other defects [29]. The dose rate range currently used for radiotherapy, including for example flattening filter free beams is approximately 1–10 Gy/min at isocentre. The dose rate at the CIED location is generally at least 10 times lower if the CIED is not located in a direct beam, i.e., lower than 1 Gy/min. Thus, in general, dose rate effects do not seem frequent in radiation therapy and, based on the theoretical failure mechanism, dose rate effects are temporary and reversible [18]. Based on the same theoretical failure mechanism, the pulse dose rate might actually be much more important, as CIED effect occurs due to the radiation-induced photocurrents generated by high dose rate and begins to be appreciable for dose rates > 10^4^ Gy/sec. In radiation therapy, we are dealing with relatively low radiation pulse dose rates (< 100 Gy/sec) so at these levels there is no significant dependence on dose rate [18]. Direct exposure from kV or MV imaging for treatment field verification occurs at often even lower dose rates. Although CIED effects have for example been shown during kV imaging (CT scanning), these effects were also temporary and reversible [29].

### In vitro studies: Cumulative dose and neutron dose

A number of in vitro studies have been published [18,25,26,28,30,31]. All of these studies show an increase in defects with an increase in accumulated dose. We have not found any evidence in the literature that the fraction size by itself is of clinical importance. Unfortunately, a reliable lower dose threshold at which no defects occur can not be established using these data because defects at very low dose levels were reported. Most probably, effects observed at very low dose levels are caused by neutrons evoking changes in the memory or the logical circuits of the CIED [32-35]. The fast and thermal neutron dose of a 18 MV beam was measured to be approximately 10–20 times higher than for a 10 MV beam in a setup build to measure the influence of radiation therapy on CIEDs [35]. Mouton et al. (2002) reported changes in the output of CIEDs at a cumulative dose to the CIED of 0.15 Gy in an 18 MV beam. They also found that cumulatively 6 and 14 of the 96 pacemakers showed a first, as they stated “important” defect at 2 Gy and 5 Gy, respectively. These numbers seem considerable. However, the authors note that some of the “important” defects reported have no clinical consequences.

### Clinical studies

Only 4 substantially sized in vivo studies have been published. Ferrara et al. reported no problems in a cohort of 45 patients with an average maximum CIED dose equal to 2.5 Gy for patients treated in the head & neck area and equal to 1.8 Gy for patients treated in the thoracic area [36]. Kapa et al. reported on 12 patients treated between 2002 and 2007 [31]. Four patients receiving radiotherapy for a tonsil tumour or left-sided lung tumour, had their CIED relocated before treatment. No CIED problems were observed in any of the reported patients. In the same paper, they reported that they did not find any defects during an in vitro study of 20 ICDs irradiated to a dose of 4 Gy. Wadasadawala et al. reported on 8 pacemaker patients receiving a cumulative dose of 0.14-60 Gy to the pacemaker and found no defects with a median follow-up of 5 months [3]. Gelblum et al. reported on 33 patients with an ICD, with dose to ICD ranging from 1 cGy to 300 cGy [37]. One patient experienced a reset of the ICD to its factory setting, being treated for rectal cancer using 15 MV photon beams. The report was initiated after they had discovered a similar reset to factory settings for a patient treated for prostate cancer using 15 MV photon beams. They suspect both resets are caused by neutrons. There are numerous case reports, of which some are included in Table 1 The details of the radiotherapy and dose levels at which defects were seen were often not clearly documented in these studies [19,27,38-40].

### Dose calculations and measurements

It is important to realise that dose levels to CIEDs reported in the literature are predominantly based on estimated values from simulator or planning data and not on direct measurements. Moreover, none of the articles report the use of heterogeneity corrections to correct for the density of the CIED itself. It is important to realise that the accuracy of the measurement or calculation only needs to be high enough to determine in which risk category the patient will fall. If one is not able to achieve this, the patient should be categorised in the highest category of the two categories that might be applicable for that patient. To within a few centimetres from the field edges, treatment planning systems generally can be used for this purpose. At a further distance, Monte Carlo based calculations are a viable tool. However, such models need to be properly modelled and validated for this purpose. Instead of calculating the dose to the CIED, one could consider measuring the dose, preferably using TLDs or OSLDs. However, accurate measurement of the dose to the CIED is not always possible [8].

### Clinical consequences of CIED malfunction

Consequences of CIED malfunction depend on the specific type of malfunction and on the pacing dependency of the patient. As described above, CIED malfunctions that have been reported are among others loss of pacing, very rapid pacing and oversensing. A detailed categorisation of effects is for example given by Hurkmans et al. [26].

### Pacing function

CIEDs are implanted for a variety of indications. Pacemakers are typically implanted for bradycardias whereas ICDs are implanted in patients with an increased risk of potentially lethal, ventricular arrhythmias. ICDs have a pacemaker function as well. Depending on the intrinsic heart rhythm of the patient and the setting of the pacemaker function, pacing by the pacemaker or ICD may occur occasionally or continuously. In a mixed population, around 10% (range: 2-63%) of patients are so-called pacing dependent, meaning they have no intrinsic or escape rhythm and may become symptomatic (syncope, arrhythmia, serious injury or even sudden death) when the CIED pacing function fails [41-43]. It is obvious that a complete loss of pacing ability will have major implications for pacing dependent patients. If the underlying heart rhythm is not sufficient, the patient will require cardiopulmonary resuscitation. Often, this is followed by external pacing through intravenously placed leads or external electrodes connected to a temporary external pacemaker. Other forms of CIED malfunction may also lead to serious problems for these patients [44]. With very rapid ventricular pacing, ventricular tachycardia may occur which can lead to a life threatening decrease of blood pressure [27]. The patient may experience palpitations, vertigo and/or syncope. If the arrhythmia deteriorates into ventricular fibrillation this may lead to the patient’s death. The same may happen when the shock function of an ICD is malfunctioning.

### Tachy-arrhythmia ICD therapy

The nature of ICD therapy is completely different. Practically all the time, the ICD acts as a watch-dog since tachycardias that may be terminated by the ICD occur infrequently. Furthermore, the moment of therapy is unpredictable. In a group of patients with ischemic cardiomyopathy in whom an ICD was implanted because of earlier ventricular arrhythmias, during 8 years follow-up, 47% of patients experienced at least one ventricular arrhythmia triggering device therapy. When one would assume that the chance of this therapy is constant, ICD therapy occurring at least once during a 6 week course of radiation treatment can be calculated at approximately 0.7%. Deactivation of the ICD shock function during the entire radiation therapy period would lead to a similar chance of withholding a potentially lifesaving shock. This is an unacceptably high figure and thus, deactivation of the ICD for an entire course of radiation to prevent inappropriate ICD therapy in radiotherapy clinical practice is not desirable. In contrast, it is shown in vitro that an ICD could interpret radiation therapy induced signals as an arrhythmia which may lead to inappropriate shock delivery [25]. Such ICD shocks in patients are uncomfortable [24,45,46], although not lethal. In the ICD population, approximately 10-20% of patients experience an unnecessary shock within a 5 year follow-up period. It has been reported that these patients have a loss in their quality of life and may develop psychological complaints as a result [47]. When possible, measures should be taken during radiotherapy to prevent an inappropriate shock delivery. This may be achieved by reprogramming of deactivating the ICD or applying a heavy magnet (90–130 Gauss). Whether or not this procedure in itself has an influence on the patient’s well-being is not known and might be subject to further study.

### Possibility of CIED relocation

In order to avoid radiotherapy related CIED problems, relocation may be considered. This may be the case when the CIED is near the volume to be irradiated. CIEDs are usually implanted in the pectoral area, with leads leading through a local vein to the heart. Relocation usually implies explantation of the CIED from the ipsilateral side and implanting a new CIED, including new leads, on the contralateral side. Leads at the ipsilateral side would typically be left in place, because of the risk of lead extraction. According to Dutch guidelines, lead removal is only performed in specialized centres with thoracic surgery at hand as 1-2% of patients will experience serious complications (0.3% mortality) requiring acute thoracic surgery [48].

The risk of CIED re-implantation at the contralateral side is equivalent to a new CIED implantation. The main risks are infection (0.4% – 4.0%) which necessitates removal of the device , and pneumothorax (0.8%-1.7%) [49]. In case of infection, relocation possibilities would be limited. In case radiotherapy may lead to CIED malfunction, an elective replacement may be warranted, which may lead to complications as well. In a large multicentre study of 2915 patients undergoing ICD replacement, 5.8% experienced a serious complication [50]. Other studies have shown that the chance of infection increases with subsequent CIED replacement [51].

### Patient perspective

It is very important to consider the patients perspective when decisions regarding the management of their CIED and treatment plan are being made. Our patient focus group consisted of 5 patients: 3 receiving treatment for prostate cancer, one for breast cancer and one for lung cancer. It was very difficult to even arrange such a small focus group, as the number of patients alive and willing to contribute to such a group is inevitably low. They unanimously considered CIED management problems related to radiation therapy of low interest as they were dealing with much more serious health care related problems. Some appreciated receiving detailed information whereas others did not wish to be informed. Collectively they all agreed that the treating radiation oncologist and cardiologist should together decide and present the best course of individualised treatment to them. It should be clear that this group of patients is too small to draw any hard conclusions. However, we hope it might stimulate a broader investigation into the expectations and needs of these patients in regard to this topic.

### Patient management

The risk of malfunction generally increases with dose and malfunction may especially be acutely deleterious in pacing dependent patients. Therefore, a patient risk categorisation is proposed incorporating these two parameters (Table 2). Sundar et al., [8] suggested a similar categorisation, making the distinction between pacing dependent and independent patients. Our new categorisation below 2 Gy is identical to the categorisation proposed by Sundar. Above 2 Gy, Sundar et al. only categorised the pacing dependent patients. We have also defined the category of pacing independent patient receiving a dose of more than 2 Gy and extended the classification for both pacing dependent and independent patients with another dose level (10 Gy). Another important distinction is their suggestion to pursue alternative treatment options or consider relocation of the CIED for medium and high risk patients. We found the advantages of these alternatives very often do not outweigh the reduction of the chance on potential CIED problems related to radiotherapy. As there are no ample data that support deviation from the 2 Gy dose level as proposed by the AAPM guideline from 1994 to distinguish risk categories, we have adopted this level here too. For the majority of patients the CIED dose will be lower than 2 Gy. A very small subgroup of around a few percent of patients with a CIED receiving radiotherapy in The Netherlands will have an estimated CIED dose above 10 Gy. Although the chances of malfunction obviously do not markedly increase above 10 Gy, this limit is considered to be practical and gives some guidance for patients which might receive a very high CIED dose. There was consensus that it would not be needed nor feasible to either adapt the treatment, relocate the CIED or ECG monitor all patients that receive a CIED dose of more than 2 Gy every fraction. On the other hand, we agreed this approach would be needed for patients receiving a much higher CIED dose. The limit was chosen to be 10 Gy rather than a higher value because the number of patients that will fall in the category of 10 Gy or higher was deemed manageable. For these patients, adaptation of the radiation therapy treatment or relocation of the CIED will often be the best choice. In exceptional cases, patients from this subgroup may receive radiation therapy with the CIED in place. This decision can only be taken in concordance with the patient and the treating cardiologist. For patients that will receive an estimated CIED dose between 2 and 10 Gy, relocation of the CIED might be considered. However, with some additional safety measures these patients might also receive radiation therapy.

**Table 2:** **Patient risk categories**: **cumulative dose to the CIED and pacing independent versus pacing dependent**

	<**2 Gy**	**2**-**10 Gy**	>**10 Gy**
pacing-independent	Low risk	Medium risk	High risk
pacing dependent	Medium risk	Medium risk	High risk

Risk defined from the patients’ perspective; how high is the risk for the patient? The patient’s risk is not equal to the risk of a CIED defect.

### Prior to radiation therapy

**Figure 1:**
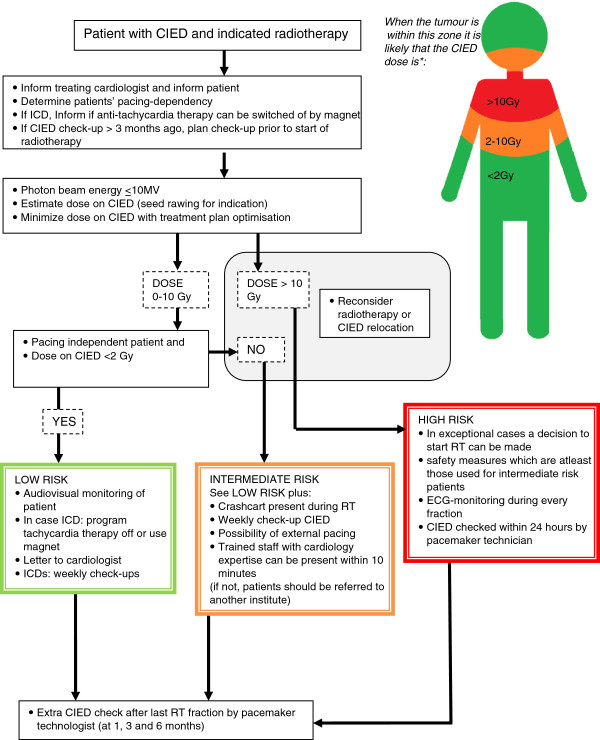
**Flow diagram of Dutch guidelines.** *Estimation of dose in case of a pectoral placed CIED.

As the specialist that considers referring a patient for radiotherapy and the treating cardiologist might not be able to very roughly estimate the possible CIED dose that can be expected to result from radiotherapy, a figure has been generated which gives upper limit estimates (Figure 1). If the patient is referred for radiotherapy, a more accurate estimation of the CIED dose should be made by the responsible clinical physicist, which may be supported by a measurement or calculation. The accuracy needs to be high enough to reliably determine the patient risk category. No heterogeneity correction for the density of the CIED should be made, as this has not been done in the vast majority of articles published about CIED dose in relation to CIED defects. The CIED patient should be identified and important information from their medical file should be available. Patients should be instructed to report any temporary or permanent cardiac symptoms that arise. To establish CIED functionality prior to treatment and to detect a possible change in the pacing-dependency of the patient, it is recommended to examine technical CIED function if this has not been done within the past 3 months (routine CIED checks are usually performed every 3–6 months). In cases in which radiation treatment is required urgently, a decision regarding the management of the patient must be made using the available information. As the risk of CIED malfunction increases with cumulative dose, the dose to the CIED should be limited as much as possible during treatment planning. Beam energies above 10 MV should be avoided due to their high neutron production, as e.g., already suggested by Gelblum et al. [37].

### During radiation therapy

The intensity of patient monitoring during radiation therapy has been adjusted to the risk of CIED malfunction and the clinical consequences. For each risk category, general measures form the baseline standard of care.

### General measures

Treating staff should be knowledgeable in the management of the patient in case complications to the CIED occur during treatment. Audiovisual observation of the patient during radiation is mandatory. In case CIED malfunction is suspected, the cardiologist and pacemaker technician need to be consulted and together with the treating radiation oncologist and the responsible clinical physicist, further management of the patient should be determined. In case of an emergency caused by a CIED defect, the standard resuscitation protocol of the institute should be followed. In case of ICDs, in order to avoid distress that may be caused by unnecessary ICD shock therapy, this should be deactivated during treatment sessions through programming or the use of a heavy magnet. If an intra-cardiac electrogram during the first fraction does not show aberrations or morphology that would trigger antitachycardia therapy, than it might be concluded that the risk of an inappropriately delivered therapy in subsequent fractions is acceptable. In this case it might be considered to refrain from deactivating the anti- tachycardia therapy during subsequent fractions. However, a careful study following every fraction is recommended.

### Low risk

The low risk group is the group of patients that receive a CIED dose below 2 Gy and are not pacing dependent. No extra measures other than the general measures are needed for this group.

### Medium risk

The medium risk group is the group of patients that receive a CIED dose of 2 Gy to 10 Gy together with the group of patients that are pacing dependent and receive a CIED dose below 2 Gy. By taking appropriate additional safety measures, this group may still be safely treated. These measures are: weekly CIED check by a pacemaker technician and adequate equipment present during each fraction, consisting of ECG-monitor and ‘crash cart’ including defibrillator or AED. Furthermore, in case of an emergency the following should be available for the pacing dependent patient: external pacing equipment (e.g. external pacemaker), personnel trained in resuscitation and a pacemaker technologist and/or cardiologist must be able to reach the patient within 10 minutes of a request in case of an emergency.

### High risk

The high risk group is the group of patients that receive a CIED dose of 10 Gy or more.

If the CIED dose is estimated to exceed 10 Gy and CIED relocation or adaptation of the radiation treatment schedule is not possible, the question should be answered whether the indications for radiation therapy outweigh the CIED related risks. If so, individual measures that are at least equal to the measures of the medium risk category must be taken. Besides, one can include ECG-monitoring during each treatment session with knowledgeable personnel present to interpret the ECG. The CIED must then be checked within 24 hours of each treatment session by a pacemaker technician.

### After radiation therapy

Several reviews recommend follow-up checks with a cardiologist because radiotherapy induced CIED defects may present long after the radiation therapy treatment course has finished. The standard frequency of CIED checks is 3–6 months, however we recommend follow-up in the cardiology department at: one, three and six months post radiation therapy treatment.

### Staff and department requirements

At least one radiation oncologist and clinical physicist with sufficient CIED knowledge should be available to ensure the correct and safe management of the patient about to undergo radiation therapy treatment. Co-operation with a cardiologist and pacemaker technician or the cardiology department is necessary as well as sufficient opportunity for ad hoc consultancy. The radiation therapists should receive specialty training for the management of CIED patients. They must know the institution specific guideline for management of patients with a CIED referred for radiotherapy as well as being able to recognise and manage CIED related problems. The radiation therapy department is responsible for training the therapists as well as ensuring the availability of specialty equipment (Table 3). When an ICD patient undergoes radiotherapy the radiation therapist must switch the ICD antitachycardia therapy off with a heavy magnet (90–130 Gauss) or a properly trained pacemaker technician must program this function off and on at each fraction.

**Table 3:** Staff and departmental requirements

	**Low risk**	**Medium risk**	**High risk**
Department	- resuscitation protocol	- see low risk+	- see medium risk+
	- good consultancy agreement with cardiology / electrophysiology dept.	- Crash cart including ECG monitor and defibrillator (or AED) available at treatment unit	- ECG monitoring at every fraction
		-external pacemaker available	
Staff	- Radiation oncologist and clinical	- see low risk+	- see medium risk+
	physicist available with sufficient	- cardiologist/pacemaker	
	knowledge in the management of patients with a CIED.	technician should be available within 10 minutes	- trained staff examines ECG
	- Radiation therapy technologists should receive training so they can manage complications experienced by the CIED patient having radiation treatment	if needed - pacemaker technologist to check CIED weekly	- pacemaker technologist checks CIED after every fraction

## In conclusion

An evidence based consensus guideline for the management of patients with a pacemaker or implantable cardioverter defibrillator has been developed. As it takes into account modern radiotherapy techniques, modern CIED technology and practical aspects for the management of these patients, it is expected to find clinical acceptance outside The Netherlands.

## Competing interests

The authors declare that they have no competing interests.

## Authors’ contributions

All authors participated in the task group that produced the Dutch guideline. CH chaired the task Group and CH and LvE drafted this manuscript. All authors contributed to the optimization of the paper and read and approved the final manuscript.
